# Diversity of tRNA Clusters in the Chloroviruses

**DOI:** 10.3390/v12101173

**Published:** 2020-10-16

**Authors:** Garry A. Duncan, David D. Dunigan, James L. Van Etten

**Affiliations:** 1 Nebraska Center for Virology, University of Nebraska-Lincoln, Lincoln, NE 68583-0900, USA; gduncan@nebrwesleyan.edu (G.A.D.); ddunigan2@unl.edu (D.D.D.); 2 Department of Plant Pathology, University of Nebraska-Lincoln, Lincoln, NE 68583-0833, USA

**Keywords:** tRNAs, tRNA clusters, chloroviruses, algal viruses, codon usage bias (CUB)

## Abstract

Viruses rely on their host’s translation machinery for the synthesis of their own proteins. Problems belie viral translation when the host has a codon usage bias (CUB) that is different from an infecting virus due to differences in the GC content between the host and virus genomes. Here, we examine the hypothesis that chloroviruses adapted to host CUB by acquisition and selection of tRNAs that at least partially favor their own CUB. The genomes of 41 chloroviruses comprising three clades, each infecting a different algal host, have been sequenced, assembled and annotated. All 41 viruses not only encode tRNAs, but their tRNA genes are located in clusters. While differences were observed between clades and even within clades, seven tRNA genes were common to all three clades of chloroviruses, including the tRNA^Arg^ gene, which was found in all 41 chloroviruses. By comparing the codon usage of one chlorovirus algal host, in which the genome has been sequenced and annotated (67% GC content), to that of two of its viruses (40% GC content), we found that the viruses were able to at least partially overcome the host’s CUB by encoding tRNAs that recognize AU-rich codons. Evidence presented herein supports the hypothesis that a chlorovirus tRNA cluster was present in the most recent common ancestor (MRCA) prior to divergence into three clades. In addition, the MRCA encoded a putative isoleucine lysidine synthase (TilS) that remains in 39/41 chloroviruses examined herein, suggesting a strong evolutionary pressure to retain the gene. TilS alters the anticodon of tRNA^Met^ that normally recognizes AUG to then recognize AUA, a codon for isoleucine. This is advantageous to the chloroviruses because the AUA codon is 12–13 times more common in the chloroviruses than their host, further helping the chloroviruses to overcome CUB. Among large DNA viruses infecting eukaryotes, the presence of tRNA genes and tRNA clusters appear to be most common in the *Phycodnaviridae* and, to a lesser extent, in the *Mimiviridae*.

## 1. Introduction

Viruses rely on most or all of their host’s translation machinery to synthesize their proteins. A conflict for viruses occurs when they have a codon usage bias (CUB) different from their host. However, some viruses have genes that help adapt to the host’s CUB in favor of their own CUB by encoding tRNAs. These include some large dsDNA viruses infecting eukaryotic organisms (see Morgado and Vicente, 2019 [1], for an extensive list) and bacteriophages [1,2]. Recently, tRNA-encoding genes have also been reported in some small ssDNA and ssRNA viruses [1].

Among the group of tRNA-encoding viruses are large viruses that infect algae [3,4,5,6], including the chloroviruses (family *Phycodnaviridae*) with a lytic lifestyle [7,8,9,10,11,12,13]. The chloroviruses have genomes that are 290 to 370 kb in size and are predicted to encode up to 400 proteins (CDSs) and 16 tRNAs [14]. They infect certain chlorella-like green algae that live in a symbiotic relationship with protists and metazoans (referred to as zoochlorellae), forming the holobiont. There are four known clades of chloroviruses based on the host they infect: viruses that infect *Chlorella variabilis* NC64A (referred to as NC64A viruses), viruses that only infect *Chlorella variabilis* Syngen 2–3 (referred to as Osy viruses), viruses that infect *Chlorella heliozoae* SAG 3.83 (referred to as SAG viruses), and viruses that infect *Micractinium conductrix* Pbi (referred to as Pbi viruses).

The genomes of more than 40 chloroviruses, representing 3 of the 4 clades, have been sequenced, assembled and annotated [13], and references cited therein. (The Osy-virus clade was not included in these analyses because at the time of this analysis few had been sequenced and annotated.) The genome GC content of the NC64A viruses ranges from 40–41%; the GC content of the Pbi viruses ranges from 44–47%, while the GC content of the SAG viruses ranges from 48–52% [13]. Their hosts, *C. variabilis* NC64A [15] and *M. conductrix* Pbi [16], both have nuclear genome GC contents of 67%, and it is assumed that *C. heliozoae*, which has not been sequenced, has a similar GC content.

Two NC64A chloroviruses, PBCV-1 and CVK2, were reported to have the interesting property of clustered tRNA genes [8,9]. Clusters of tRNA genes are found in a small percentage of organisms from all three domains of life [1,17,18,19]. tRNA-gene clusters have also been reported to occur in mitochondria and plastids [2,20,21]. In terms of viruses, Morgado and Vicente [1] recently reported that tRNA genes were in ~14% of the 13,200 virus genomes that they examined and that the tRNAs occurred in clusters in 1.7% of the tRNA encoding viruses, many of which were bacteriophage. Therefore, clusters of tRNAs encoded by viruses that infect eukaryotic organisms are not very common.

This current in silico investigation had several purposes: (i) To evaluate chlorovirus tRNA genes with respect to clustering. (ii) To compare and contrast the tRNA genes within and among the three clades of chloroviruses. (iii) To determine if there was a CUB in the algal host *C. variabilis* NC64A, and likewise, if there was a CUB among the chloroviruses. iv) To evaluate whether the chlorovirus clades acquired their tRNA gene clusters before or after their divergences from their most recent common ancestor (MRCA). (v) To investigate the role that tRNA isoleucine lysidine synthase, an enzyme encoded by almost all chloroviruses, might play in overcoming CUB. (vi) To examine the incidence of tRNA genes and gene clustering within other members of the *Phycodnaviridae* and in some other large DNA viruses.

## 2. Materials and Methods

### 2.1. Genomic Sequence Data

In total, the genomes of 41 sequenced, assembled (in some cases to draft genomes) and annotated chlorovirus genomes that represent three of the four known chlorovirus clades were used in this study: 14 NC64A viruses, 13 SAG viruses, and 14 Pbi viruses ([13] and references cited therein). Table S1 provides the accession numbers, as well as the collection source of the viruses. The genome of *C. variabilis* NC64A, which is the host of the NC64A viruses, was previously sequenced and annotated [15] (project accession number ADIC00000000). *M. conductrix*, the host of the Pbi virus, has also recently been sequenced and annotated [16], but it was not used in this study.

### 2.2. Identification and Localization of the tRNA Genes 

A tRNA gene cluster was previously reported for the chlorovirus PBCV-1 [9,22], as well as chlorovirus CVK2 [8]. The other 40 chloroviruses were examined herein to determine if they had tRNA gene clusters, and their tRNA genes and gene order were also documented. The tRNA genes for each virus were identified by entering the genome accession numbers into the Nucleotide database at NCBI (ncbi.nlm.nih.gov). The Graphics link was used to identify the tRNA genes and their locations, as well as the surrounding non-tRNA genes. The putative tRNA sequences were verified using tRNAscan-SE [23,24]. Commonalities among the three clades of chloroviruses, as well as their differences, were also recorded. tRNA gene information for other nuclear cytoplasmic large DNA viruses (NCLDVs) was obtained from the NCBI database and from Table S2 from Morgado and Vincente [1].

For the chloroviruses examined herein, the non-tRNA genes immediately 5′ and 3′ to the tRNA clusters were sought in order to give insight as to whether the 41 chloroviruses acquired their tRNA gene clusters prior to or after their divergence into the three clades. The source of the data was the same NCBI website, and the data were processed as described above.

### 2.3. CUB in Chloroviruses and Their Host

Two NC64A viruses, PBCV-1 and AN69C, were selected to compare their codon usage to that of their host, *C. variabilis* NC64A using Geneious 11.1.5 (Biomatters Ltd., Auckland, New Zealand, https://www.geneious.com).

### 2.4. Phylogenetic Tree Construction of tRNA Genes

Three concatenated tRNA genes common to all three chlorovirus clades were analyzed by phylogenetic analysis: tRNA^Tyr^, tRNA^Gly^, and tRNA^Arg^. The following chloroviruses were not included in this analysis because they lacked one of the three tRNA genes that were concatenated: PBCV-1, NE-JV-1, AR158, NE-JV-4, GM0701.1, and MN0810.1. The tRNA sequences were retrieved from NCBI. The concatenated sequences were aligned by MUSCLE and curated by the Gblocks method; phylogenies were constructed using three tree building algorithms that included 100 bootstraps each: maximum likelihood, parsimony and BioNJ, a distance method (http://phylogeny.fr). The trees were saved in Newick format and MEGAX was used to present the final trees [25]. Phylogenetic trees using each of the three individual tRNA genes were also constructed by the procedures described above.

## 3. Results and Discussion

### 3.1. Chlorovirus tRNA Gene Clusters

All 41 chloroviruses encode tRNA genes, and the tRNA genes were located in clusters, usually with intergenic spacers of 1 to ~30 nucleotides (Table 1, Table 2 and Table 3). However, in a few cases non-tRNA genes were also present within the tRNA clusters, resulting in larger spacers between contiguous tRNA genes. Collectively, the 41 chloroviruses encoded a total of 410 tRNAs of which there were 17 different tRNAs for 14 different amino acids (3 synonymous codons).

The 14 NC64A viruses had from 7 (virus AR158) to 14 (viruses MA-1E, CvsA1, and CviK1) tRNA genes in their clusters (Table 1); the 13 SAG viruses had from 7 (NTS-1) to 13 (OR0704.3, Can0619SP, NE-JV-2) tRNA genes in their clusters (Table 2); and, the 14 Pbi viruses had from 3 (NE-JV-1) to 11 (Fr5L) genes in their tRNA gene clusters (Table 3). If one assumes that the original clusters are the sum of all the tRNAs genes in a clade, the NC64A chlorovirus cluster would consist of 14 tRNA genes; indeed, 3 of the NC64A viruses had all 14 tRNA genes. However, the sum of the SAG viruses was 18 tRNA genes, but the largest number of tRNA genes in any one extant virus was 13; the sum of the Pbi viruses was 15 tRNA genes, but the largest number of tRNA genes of any one extant virus was 11 tRNA genes. Some of the tRNA genes likely represent gene duplications. For example, all 41 chloroviruses had 2–4 tRNA^Asn^ genes, in which the encoded tRNAs recognize the same codon AAC.

The order of the chlorovirus tRNA genes within a cluster is also reported in Table 1, Table 2 and Table 3. In general, there was synteny of tRNA genes among the viruses within a clade, but not between clades. (Exceptions to synteny within a clade are noted in the footnotes to Table 1, Table 2 and Table 3.) Seven tRNA genes were common to one or more members of all three chlorovirus clades, although not present in every virus isolate within any of the three clades (Table 4). Two tRNA genes were unique to the NC64A viruses, 3 tRNA genes were unique to the Pbi viruses and 4 tRNA genes were unique to the SAG viruses. Three additional tRNA genes were common to one or more members of the NC64A and SAG clades. The tRNA^Arg^ gene was found in all 41 chloroviruses, while 39/41 chloroviruses had the tRNA^Gly^ gene.

Due to the short intergenic spacers between clustered chlorovirus tRNA genes, we suspected that the tRNA gene clusters were transcribed as one transcript. Indeed, Nishida et al. [8] reported that chlorovirus CVK2 transcribed its tRNA gene cluster of 14 tRNAs into one transcript; furthermore, the RNA transcript was precisely processed into individual tRNA species by either some unknown virus-encoded or host-encoded RNase. In this regards, PBCV-1 encodes a functional RNase III enzyme [26], but its role in virus replication is unknown.

Differences and similarities in tRNA gene content were observed among individual chloroviruses within each of the three clades of chloroviruses. In some chloroviruses, not all of the tRNA genes within a gene cluster were immediately contiguous to one another, leading to interrupted tRNA-gene clusters in which one or several non-tRNA genes were interspersed within the tRNA-gene cluster. This was especially true for the Pbi viruses in which at least four member viruses had interrupted tRNA-gene clusters, as indicated by large nt intergenic spacer numbers in Table 3.

Additionally, all of the chlorovirus-encoded tRNAs lacked the 3′ terminal three nucleotides (CCA) necessary to be aminoacylated. Since fully functional tRNAs were reported for the NC64A chlorovirus CVK2 [8], the chlorovirus tRNAs, like tRNAs from cellular organisms [27], must either use an unidentified virus enzyme(s) or the host tRNA nucleotidytransferase to add the CCA nucleotides prior to tRNA aminoacylation.

### 3.2. Genome Location of the Chlorovirus tRNA Clusters

The tRNA clusters in the NC64A and Pbi viruses were located near the center of their genomes except for the Pbi virus NE-JV-1, which was located in the last third of its genome; NE-JV-1 is unusual in many other aspects including the fact that it only has 3 tRNA genes [13]. The tRNA clusters in all of the SAG viruses were located in the first third of their genomes. It is interesting to note that all 13 SAG viruses had a tRNA^Thr^ gene located ~30 kb beyond the 3′ end of the tRNA gene cluster, placing the tRNA^Thr^ genes near the center of their respective genomes.

One possible explanation is that the tRNA cluster in the SAG viruses was translocated in the 5′ direction but without the tRNA^Thr^ gene in the SAG clade’s MRCA. Indeed, if a translocation event did take place, then the original location of the cluster in the ancestral SAG virus would have been more towards the center of its genome. In support of this notion, all of the Pbi viruses have a tRNA^Thr^ at the 3’ end of their respective clusters.

### 3.3. Potential tRNA Transcription Promoters

As noted above, Nishida et al. [8] reported that the tRNA gene cluster from chlorovirus CVK2 was transcribed as a single RNA, which was then processed to the individual tRNAs. RNA polymerase III utilizes type 2 (Class II) promoters to begin transcription of tRNA genes, individually or in clusters [28,29,30]. Type 2 promoters consist of two 11-mer intragenic sequences referred to as Box A and B, which are first recognized by transcription factor IIIC (TFIIIC) that begins the cascade of events leading to the attachment of RNA polymerase III. The chlorovirus tRNA genes have these two boxes, as do the 13 orphaned tRNA^Thr^ genes in all 13 SAG viruses located approximately 30 kb downstream of their respective tRNA clusters (Figure S1). This suggests that the tRNA^Thr^ genes in the SAG viruses are transcribed independently of their respective tRNA clusters.

### 3.4. Presence of CUB Differences in Host and Viruses

Because nuclear genomic codon usage data are available for *C. variabilis* NC64A and its viruses, we were able to compare their respective codon usages (Table 5). Codon usage was available for 13/14 NC64A viruses, but only two representative NC64A viruses, PBCV-1 and AN69C, were chosen for the comparative study. CUB favoring codons with high GC content were noted in the host alga *C. variabilis* NC64A whose genome is 67% GC (Table 5), while the two NC64A viruses have GC contents of 40% [13] and a CUB favoring AU (Table 5). For example, in the standard universal code there are four codons for the amino acid alanine, differing only in the third (3′) base of the codon. Additionally, in the standard universal code there are four codons for the amino acid glycine, differing only in the third (3′) base of the codon. In *C. variabilis* NC64A, the two codons whose third base was C or G (GGC and GGG) were the most common, while the two most common in PBCV-1 and AN69C ended in A and U (GGA and GGU) (Table 5). A parallel example occurs in the usage of the two synonymous codons for glutamic acid; GAG was almost exclusively used by *C. variabilis* NC64A, while GAA was the most common in the two NC64A viruses. The same was true for all amino acids encoded by two synonymous codons (asparagine, aspartic acid, cysteine, glutamine, histidine, lysine, phenylalanine and tyrosine), as well as other amino acids with more than two synonymous codons. Furthermore, the isoleucine codon AUA (AU-rich) occurred in 2.44% of all PBCV-1 codons, while it occurred in only 0.20% of codons in *C. variabilis* NC64A, a 12-fold difference (Table 5); hence, in the virus, natural selection appears to have favored the retention of the gene that encodes the cognate tRNA for this codon. Likewise, the lysine codon AAA (AU rich) occurred in 4.7% of all PBCV-1 codons, but in only 0.25% of host codons, nearly a 20-fold difference. The leucine codon UUA was the rarest codon used by *C. variabilis* NC64A (0.06% of all codons), while it was a moderately common codon used by PBCV-1 (1.39% of all codons) (Table 5). As a final example, there are six synonymous codons for the amino acid arginine, but only one codon that had one guanine or cytosine (AGA), while the other five codons had a minimum of two guanines and/or cytosines. 1.43% of all viral codons were AGA for arginine, but only 0.25% of *C. variabilis* NC64A codons were AGA. Indeed, the two arginine codons with three guanines and cytosines (CGC and CGG) were the two most common in *C. variabilis* NC64A (3.22% and 2.11%, respectively), while the same two codons in the two viruses were 0.75% and 0.70%, respectively.

### 3.5. Not All Chlorovirus tRNAs Assist in Overcoming CUB

The CUB of the host is overcome by some but not all tRNA members in the viral tRNA clusters. For example, PBCV-1 encodes 11 tRNAs but only 7 help it to overcome CUB; likewise, AN69C encodes 10 tRNAs but only 8 favor its own CUB (Table 5). Thus, we speculate that the cluster as a unit is under natural selection, rather than each individual tRNA gene, because some of the tRNA genes in the clusters are neutral and do not bestow any positive selective advantage. A reasonable explanation for these observations is that the neutral tRNA genes are unvetted by natural selection, acting as hitchhikers due to their close linkage to tRNA genes that help the viruses overcome the CUB of the host. To illustrate this point, all three clades of chloroviruses had 2–4 tRNA^Asn^ genes whose tRNAs only recognize the codon AAC; none of the 41 chloroviruses encode a tRNA^Asn^ that recognizes the alternate codon AAU, which was 10 times more common in the two viruses than in *C. variabilis* (Table 5), and which would presumably enhance viral protein translation. Of no surprise, the most common of the two Asn codons in *C. variabilis* is AAC.

The presence of the AAC codon cognate tRNA^Asn^ genes, which appears to be neutral in benefit to all 41 chloroviruses, suggests that they were acquired in the MRCA tRNA cluster due to an evolutionary accident. That is, their presence appears to be an artifact of evolutionary history by which some neutral tRNA genes were acquired in the tRNA cluster along with selectively advantageous genes by some mechanism, such as horizontal gene transfer (HGT). (This kind of event is similar to the frozen accident concept first proposed by Crick [31].) In the case at hand, neutral tRNA^Asn^ genes may be maintained in the tRNA gene clusters seemingly due to their tight linkage to selectively advantageous viral tRNA genes in the cluster. This seems particularly conceivable if the tRNA cluster is encoded as a single RNA transcript, as it is with chlorovirus CVK2 [8]. The same argument may explain the presence in PBCV-1 of the tRNA^Lys^ gene for the cognate codon AAG. PBCV-1 encodes both of the tRNAs genes that recognize the two lysine codons, AAG and AAA. However, the AAA codon was >20 times more common in PBCV-1 than in *C. variabilis*, while AAG was the most common lysine codon in *C. variabilis*; hence, the former appears to be maintained by positive selection, while the latter appears to provide a neutral benefit to PBCV-1.

Although the tRNA analysis software (http://lowelab.ucsc.edu/tRNAscan-SE/) predicts that all of the encoded chlorovirus tRNAs are functional, with the noted exceptions of several tRNA genes whose mutations have rendered them as pseudogenes, appropriate laboratory-based verification of functionality was beyond the scope of this study. However, Nishida et al. (1999) did demonstrate that some of the tRNAs encoded by CVK2, an NC64A virus, are functional. While we think it is unlikely, we cannot rule out the possibility that some or all of the viral-encoded tRNAs discussed herein are nonfunctional.

### 3.6. Chloroviruses Encode a Putative tRNA Isoleucine Lysidine Synthase (TilS)

**Figure 1:**
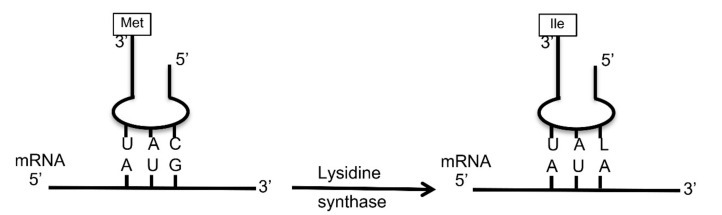
Simplified diagram of tRNA anticodon base pairing with the mRNA codon. The tRNA^Met^ anticodon 3′ UAC 5′ recognizes the codon 5′ AUG 3′ (left figure). The enzyme tRNA isoleucine lysidine synthase (TilS) attaches lysine to the 5′ cytosine of the tRNA, which becomes lysidine. Lysidine base pairs with adenine. As such, the modified tRNA now recognizes the isoleucine codon AUA.

Thirty-nine of the 41 chloroviruses encode another putative enzyme involved in codon usage, TilS. The methionine codon AUG is normally recognized by its cognate tRNA^Met^ with the 3′ UAC 5′ anticodon. The TilS enzyme ligates lysine to the cytidine in the 5′ position of the tRNA^Met^ anticodon; this modified cytidine becomes lysidine, which is complementary to adenine in the 3′ position of the codon, rather than guanine (Figure 1). As such, this modified tRNA then behaves as a tRNA^Ile^ and recognizes the isoleucine AUA codon [32,33]. The AUA codon is 12–13 times more common in the two NC64A viruses than in the host, *C. variabilis* (Table 5). Thus, we suspect this enzyme provides one additional mechanism that helps the viruses overcome CUB of the host by enabling more efficient viral protein synthesis, diminishing the chances of a ribosome stall during elongation when AUA codons are encountered. A previous PBCV-1 transcription RNA-seq study reported that the *tils* gene was transcribed very early during virus infection, detected by 7 min post infection [34].

All chloroviruses also encode a homolog of translational elongation factor 3 (EF-3) [13]. EF-3 plays a role in optimizing the accuracy of mRNA decoding at the ribosomal acceptor site during protein synthesis in fungi [35,36]; EF-3 has been reported recently in algae [37]. The role this putative enzyme plays in chlorovirus translation is unknown.

### 3.7. Acquisition of Chlorovirus tRNA Clusters

One question we addressed was: Did the three chlorovirus clades independently acquire their tRNA clusters, e.g., by HGT, or was the tRNA gene cluster acquired by the MRCA, i.e., the last common ancestor prior to splitting into the three clades? We used several lines of inquiry to address this question. First, as described above, the three clades of chloroviruses had 7 tRNA genes in common with one another (Table 4), including 2–4 tRNA^Asn^ genes, in which the encoded tRNAs recognize the codon AAC. There were, however, considerable differences in composition and order between clades, unlike the similar composition and synteny within each of the three clades (Table 1, Table 2 and Table 3).

The second line of inquiry focused on the protein-encoding genes that border the 5′ and 3′ sides of the tRNA clusters. Supplementary Tables S2–S4 report the commonness of protein-encoding genes (orthologous genes) within each of the three clades, but the protein-encoding genes surrounding the tRNA clusters of each clade were not orthologous across the three clades.

**Figure 2:**
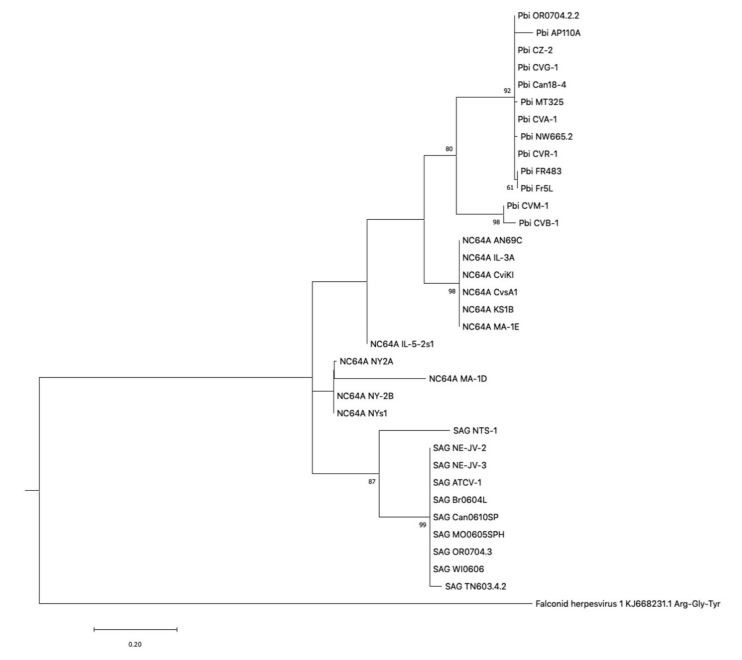
Phylogram of three concatenated tRNA genes—tRNA^Gly^, tRNA^Tyr^, and tRNA^Arg^—using the maximum likelihood tree building algorithm with 100 bootstraps using the online website: phylogeny.fr. The concatenated sequences were aligned by MUSCLE; the alignment was curated by the Gblocks program. Parsimony and distance (BioNJ) phylogenetic tree-building algorithms were also used (100 bootstraps) and produced similar tree branching topologies. The numbers on the tree represent supported bootstrap values (i.e., percentage of bootstrap replicates that produced the same tree branch). The following chloroviruses were not included in the analysis because they lacked one of the three tRNA genes that were concatenated: PBCV-1, NE-JV-1, AR158, NE-JV-4, GM0701.1, and MN0810.1.

A third line of inquiry was to examine phylogenetic tree constructs in which the three tRNA genes common to all three clades of chloroviruses (tRNA^Gly^, tRNA^Arg^, and tRNA^Tyr^) were concatenated (Figure 2). The Pbi and SAG clades clustered together, as expected. The NC64A viruses, however, formed two distinct clades. The tree is supported by the results shown in Table 1, in which there is a pattern of presence/absence of tRNAs genes that distinguishes two groups of NC64A viruses.

Phylogenetic trees were also constructed for each of the three individual tRNA genes, tRNA^Tyr^, tRNA^Gly^, and tRNA^Arg^ (Figures S2–S4, respectively). For each of the three individual tRNA gene analyses, most of the chloroviruses tended to cluster within their clade, but there were a number of exceptions, and it was the exceptions that support a single HGT event hypothesis in the MRCA. For example, the tRNA^Tyr^ genes of four of the NC64A viruses were more similar in nucleotide sequence to the SAG viruses than to other NC64A viruses (Figure S2). Likewise, for the tRNA^Gly^ gene, half of the NC64A viruses were more similar to SAG viruses, while the other half of the NC64A viruses were more similar to Pbi viruses (Figure S3); furthermore, there were two Pbi and two SAG viruses that were more similar to a subclade of NC64A viruses than to other members of their own clade.

A fourth line of inquiry focused on the predicted intron in the tRNA^Tyr^ gene found in 34/41 chloroviruses representing all three clades. We examined the locations and sequences of the introns and found that the introns were located in identical positions in all of the tRNA^Tyr^ genes, one nucleotide from the anticodon (3′ direction). While the intron lengths and sequences were nearly identical within a clade, there were slight differences in intron length between the three clades: NC64A 13–14 nt; Pbi 13 nt; SAG 10–11 nt. Because introns within tRNA^Tyr^ genes are relatively rare, it is unlikely that all of these tRNA^Tyr^ genes with introns were acquired independently. Rather, from an Occam’s razor line of inference, the evidence supports a single origin for the chlorovirus tRNA^Tyr^ gene and, therefore gene clusters, prior to the divergence of the three clades from the MRCA.

It is also noted that all 410 tRNA genes of all 41 chloroviruses are on the same identical DNA strand and none on the alternate DNA strand. Separate acquisition events over time would likely have resulted in at least some tRNA genes being acquired on the alternate DNA strand.

### 3.8. Generation of Chlorovirus tRNAs

The ability of tRNA genes to proliferate is thought to be similar to the mechanism by which mobile elements can lead to intragenomic gene duplications [38]. Duplications and losses are clearly evident among and within all three chlorovirus clades; for example, the Pbi virus CZ-2 had four tRNA^Asn^ genes that all recognized the same ACC codon, while almost all of the other Pbi viruses had just two copies. Likewise, the NC64A virus MA-1E had three tRNA^Lys^ genes that all recognized the same AAG codon, while others in the same clade had just one, and virus AR158 had none. There are other similar examples among the three chlorovirus clades.

Pope et al. [39] implicated a homing endonuclease (HNH) in the generation of tRNA genes in mycobacteriophages. While not immediately adjacent to the tRNA gene clusters, all of the chloroviruses encoded at least two putative HNHs (e.g., orthologs of A087R and A422R in PBCV-1). A second source of HNH or other endonucleases could be from co-infecting viruses with such genes in their repertoire that generated tRNA gene duplications, losses, and translocations within the chloroviruses. Previously, we proposed three potential sources of HGT for the chlorovirus protein-encoding genes that might also explain the tRNA clusters in the chloroviruses [13]: (i) viral host(s), although there are only a few NC64A chlorovirus genes that have probably been acquired from *C. variabilis* NC64A; however, the viruses probably had at least one other host through evolutionary time; (ii) bacteria, because some of the chlorovirus genes appear to be of bacterial origin; and, (iii) from other host-competing viral species. Plastids and mitochondria might also have contributed to the viruses via HGT, at least for the tRNA gene clusters, because those organelles also have tRNA gene clusters [2,20,21]. These results are consistent with the analyses and conclusions of Fan et al. [20] who sequenced the mitochondria and plastids of the three chlorovirus hosts, *C. variabilis*, *C. heliozoae*, and *M. conductrix.* Morgado and Vicente [1] suggested that viruses with tRNA clusters might be the source of dissemination of such clustered structures in the three domains of life.

### 3.9. tRNA Gene Clusters in other Phycodnavirues

Most of the other viruses in the *Phycodnaviridae* family also encode tRNAs that are in gene clusters (Table 6). *Micromonas pusilla* virus 12T (MpV12T) encodes six tRNA genes, five of which are clustered. The sixth tRNA gene, tRNA^Thr^, is an orphan ~30 kb beyond the tRNA^Leu^ gene, the 3′ member of the tRNA cluster. The position of these two tRNA genes and the distance between them is the same as in the chlorovirus SAG clade that also has a 30 kb gap between the genes that encode tRNA^Leu^ and tRNA^Thr^. MpV12T also encodes a tRNA^Tyr^, as do the chloroviruses, but unlike the chloroviruses, MpV12T does not have an intron in its tRNA^Tyr^ gene. On the other hand, *Ostreococcus lucimarinus* virus 7 (OlV7) [6], which has five tRNA genes, four of which are clustered, encodes a tRNA^Tyr^ that does have a 15 nt intron located in the same position as the chloroviruses. In fact, the tRNA genes in the *Micromonas* and *Ostreococcus* virus clusters are similar to some of the tRNA gene clusters in the NC64A viruses, suggesting the two groups of viruses might have a common evolutionary ancestor. The only phycodnavirus that lacks tRNA encoding genes is *Ectocarpus siliculosus* virus 1 (EsV1) (Table 6); however, EsV1 is unusual because it is the only phycodnavirus that has a lysogenic life style.

### 3.10. tRNA Genes and Gene Clusters in other Large DNA Viruses

Viruses in the *Phycodnaviridae* family are proposed to have an ancient, common evolutionary ancestry with some other large dsDNA viruses in the *Poxviridae*, *Iridoviridae*, *Ascoviridae*, *Asfarviridae*, *Mimiviridae*, *Marseillivirdae*, and *Pithoviridae* families that infect eukaryotes. However, many more large DNA viruses are rapidly being discovered, including Pandoraviruses, Faustoviruses, Mollivirus, Kaumoebavirus, Cetratvirus, Pacmanvirus, and Orpheoviruses, and the evolutionary relationships among these viruses are just starting to be analyzed [40,41]. Collectively, these giant viruses are referred to as NCLDVs [42,43,44].

Therefore, we examined a few representatives from all of these groups to see if they encoded tRNAs and, if so, did the tRNA genes occur in clusters (Table 7). All of the *Mimiviridae* viruses that were examined encoded tRNA genes and 8/9 had tRNA gene clusters. Most of the remaining NCLDVs, with few exceptions, encoded 0–3 tRNA genes, and none of them had 2 or more tRNA genes in clusters. A few baculoviruses and herpes viruses were also examined for tRNA-encoding genes. Nine of the baculoviruses examined encoded 1 tRNA gene. The 6 herpes viruses that were examined contained from 1 to 19 tRNA genes, but only one of the viruses had clustered tRNA genes (Table 7). Thus, the *Phycodnaviridae* viruses, along with many viruses in the *Mimiviridae* family, appear to be the exceptions among the large DNA viruses by having clustered tRNA genes.

## 4. Conclusions

The chloroviruses overcome CUB of their algal hosts by encoding clusters of tRNA-encoding genes, with most of the tRNAs favoring the viruses. However, the evolutionary events associated with the tRNA clusters differed among the viruses, even within the same clade, because very few of the tRNAs were conserved among all of the chloroviruses. In addition, 39/41 chloroviruses encode a putative enzyme that modifies a tRNA to recognize a codon that is 12–13 times more prominent in the chloroviruses than their host, further acting to curb CUB. As well, it has not escaped our attention that Wobble, which has been extensively investigated and updated, may assist viral protein synthesis. For example, the two codons for tyrosine, UAU and UAC, can be recognized by the same tRNA^Tyr^ due to Wobble at the 5′ G of the tRNA, which can base pair with the U or C at the 3′ position of the codon. Wobble can also facilitate some of the other encoded amino acids including the codons for glutamic acid, cysteine, histidine, phenylalanine, etc. All the chloroviruses examined to date have clustered tRNA genes, as do many of their *Phycodnaviridae* relatives. With the exception of *Mimiviridae* viruses, most other large DNA viruses not only have few, if any, tRNA genes, but when a virus encodes two or more tRNA genes, they are not clustered.

Finally, we are aware that tRNAs are turning out to play other roles in cells besides protein synthesis [45], so it is always possible that the viral encoded tRNAs serve some other functions.

## Figures and Tables

**Table 1:** The order of clustered tRNA genes in NC64A chloroviruses ^1^.

	Leu-1 UUG		Ile AUA		Asn-1 AAC		Leu-2 UUA		Arg-1 AGA		Asn-2 AAC		Gly GGA		Asn-3 AAC		Lys-1 AAG		Gln CAG		Lys-2 AAG		Tyr UAC ^a^		Lys-3 AAA		Lys-4 AAG		Arg-2 AGA		Asp GAC		Val GUU	Total tRNAs
**MA-1E**		25		23				24				3		23		3		27		3				22		23		23		30		1		14
CvsA1		25		23				24				3		23		3		27		3				22		23		23		30		1		14
CviK1		25		23				24				3		23		3		27		3				22		23		23		30		1		14
KS1B		3		23				109				3		23		3		27		3				22		23		23		30		1		12
PBCV-1		25		23				23				3	^b^	24		3		24						22		23				33				11
IL-3A		25		23				24				3		23		3		25	^c^	3		84		22		23								10
MA-1D		25		23				24				5		15		3		25		3				22		23				33				12
NE-JV-4		25		23				24				3		23		3		72	^c^	3						23				33				11
AN69C		25		23				24				3		23		3		12						22		23								10
NY-2B		25		23				23		3				23		22								2		25								8
IL-5-2s1		25		23				23		3				23		22								2		25								8
NY-2A		142						23		3				24		22								2		25								8
NYs-1		25		25		229				3				1058										2		25								8
AR158		25		25		51		23		3				24																				7

^1^ All tRNAs genes are within a single cluster for each of the 14 NC64A viruses. The heading of each column identifies the cognate amino acid and codon for each encoded tRNA. Green color indicates same tRNA gene as other members in the column; orange indicates a pseudogene; red indicates a tRNA gene substitution; white indicates absence of tRNA gene. The numbers in the white columns specify the number of nt between two adjacent tRNAs; a missing number in a column is due to a missing tRNA gene. ^a^ tRNA^Tyr^ contains an intron; ^b^ tRNA^Lys^ substitution for tRNA^Gly^; ^c^ tRNA^Asn^ substitution for tRNA^Gln^.

**Table 2:** The order of clustered tRNA genes in SAG chloroviruses ^1^.

SAG Viruses	Codons and Cognate Amino Acids Recognized by SAG Virus tRNAs																																							
	Ile-1 AUA		Ser AGU		Arg AGA		Asn-1 AAC		Gly GGA		Ile-2 AUU		Asn-2 AAC		Met AUG		Asp-1 GAC		Val-1 GUU		Val-2 GUU		Asn-3 AAC		Tyr UAC ^a^		Lys AAG		Asn-4 AAC		Asp-2 GAC		Leu-1 UUA		Asn-5 AAC		Leu-2 UUG		Thr ACU ^b^	Total tRNAs
**Can0610SP**				4		25				22		22		25				23		22				22		2		21		4				36k						13
OR0704.3				4		25				22		22		25				23		22				22		2		21		148				32k						13
NE-JV-2		22		4		25				22		22		22				23		22						2		21		148				29k						13
NE-JV-3				4		25				22		22		22				23		22						2		21		148				31k						12
ATCV-1				5		25				22								23		22		22		22		2		22		148			c	31k						11
WI0606				4		25				22								23		22				22		2		22		148				31k						11
MO0605SPH				4		25				22								23		22				22		2		22		148				30k						11
GM0701.1						25		1		22				24				23												148				77		4		35k		10
Br0604L				4		25				22				25				23								2		242						32k						9
TN603.4.2						25				22				24				23								2		22		227				32k						9
Canal-1		158		5		25				22						65												23		24		54		29k						9
MN0810.1				23		24		1		22										22		22		4										35k						9
NTS-1				23		25				22				21												23				33k										7

^1^ All tRNAs genes are within a single cluster for each of the 13 SAG viruses, with the exception of tRNA^Thr^, which is an orphan tRNA 29–36kb downstream of its respective tRNA cluster. The heading of each column identifies the cognate amino acid and codon for each encoded tRNA. Green color indicates the same tRNA gene as other members in the column; orange indicates a pseudogene; white indicates the absence of the tRNA gene. The numbers in the white columns specify the number of nt between two adjacent tRNAs; a missing number in a column is due to a missing tRNA gene. ^a^ tRNA^Tyr^ contains an intron; ^b^ This tRNA gene is 30,000–31,502 nt downstream of the tRNA cluster.

**Table 3:** The order of clustered tRNA genes in Pbi chloroviruses ^1^.

Pbi Viruses	Codons and Cognate Amino Acids Recognized by Pbi Virus tRNAs																													
	Ile AUA		Leu UUA		Phe UUC		Arg AGA		Gly GGA		Asn-1 AAC		Tyr-1 UAC ^a^		Lys-1 AAG		Asn-2 AAC		Asn-3 AAC		Asn-4 AAC		Tyr-2 UAC ^a^		Lys-2 AAG		Thr-1 ACG		Thr-2 ACG	Total tRNAs
**Fr5L**						23		3		22		22		2		25		22		22				2				245		11
CZ-2						23		3		22		23						23		22		22		2		243				10
MT325		24		24		23		3		23		22						22						2		161				10
Can18-4		24		24		23		3		23		22						22						2		1041				10
CVB-1		24		24		26		985		24		23						22						2		161				10
FR483		22		24				3		23		23						22						2		161				9
CVG-1		132				23		3		23		23						22						2		161				9
CVR-1		132				23		3		23		23						22						2		159				9
CVA-1		132				23		3		23		23						22						2		159				9
AP110A		132				23		3		23		23						23						2		159				9
CVM-1		132				23		3		24		96						22						2		161				9
NW665.2		1142						3		23		23						22						2		161				8
OR0704.2.2								3		23		22						22						2		159				7
NE-JV-1		132						1416																						3

^1^ All tRNAs genes are within a single cluster for each of the 14 Pbi viruses. The heading of each column identifies the cognate amino acid and codon for each encoded tRNA. Green color indicates the same tRNA gene as other members in the column; white indicates the absence of the tRNA gene. The numbers in the white columns specify the number of nt between two adjacent tRNAs; a missing number in a column is due to a missing tRNA gene. ^a^ tRNA^Tyr^ contains an intron.

**Table 4:** Chlorovirus tRNA genes common to one another and unique to each chlorovirus clade ^1^.

tRNA	Codon	NC64A	SAG	Pbi
Ile-1	AUA	c	c	c
Leu-1	UUA	c	c	c
Asn-1	AAC	c	c	c
Gly-1	GGA	c	c	c
Lys-1	AAG	c	c	c
Tyr-1	UAC	c	c	c
Arg-1	AGA	c	c	c
Asp-1	GAC	d	d	
Val-1	GUU	d	d	
Leu-2	UUG	d	d	
Gln-1	CAG	u		
Lys-2	AAA	u		
Ser-1	AGU		u	
Ile-2	AUU		u	
Met-1	AUG		u	
Thr-1	ACU		u	
Phe-1	UUC			u
Thr-1	ACG			u
Thr-2	ACG			u

^1^ (c) means that some members in all three clades of the chloroviruses have the gene. (d) means that some members in the NC64A and SAG chlorovirus clades have the gene. (u) means that the gene is unique to some viruses in one of the three clades of chloroviruses.

**Table 5:** Codon frequency use comparison of two chloroviruses, PBCV-1 and AN69C, to *Chlorella* host.

Codon AA	PBCV-1	AN69C	*C. variabilis*	Ratio ^1^	Codon AA	PBCV-1	AN69C	*C. variabilis*	Ratio ^1^
GCA A	1.92	1.15	2.42	0.63	**AAC N**	**2.62**	**2.28**	1.48	**1.66**
GCC A	0.86	0.64	5.54	0.14	AAU N	3.16	2.78	0.30	9.9
GCG A	1.29	0.84	5.46	0.20	CCA P	1.42	1.24	1.08	1.23
GCU A	1.30	0.82	1.72	0.62	CCC P	1.07	0.91	2.55	0.39
UGC C	0.67	1.08	1.77	0.49	CCG P	0.96	0.97	2.30	0.42
UGU C	1.23	1.88	0.32	4.86	CCU P	1.33	0.89	0.96	1.16
GAC D	1.97	1.31	3.04	0.54	CAA Q	1.84	2.09	0.59	3.33
GAU D	3.02	1.91	1.06	4.56	CAG Q	0.87	1.06	4.93	0.2
GAA E	3.69	2.47	0.54	**5.70**	**AGA R**	**1.43**	**1.71**	0.25	**6.28**
GAG E	1.26	1.08	4.92	0.24	AGG R	0.68	0.84	0.92	0.83
UUC F	2.53	2.40	1.55	1.59	CGA R	0.66	1.48	0.44	2.43
UUU F	2.94	3.49	0.95	3.38	CGC R	0.66	0.84	3.22	0.23
**GGA G**	1.71	**1.28**	0.76	**1.97**	CGG R	0.45	0.94	2.11	0.32
GGC G	0.61	0.61	5.88	0.10	CGU R	1.05	1.38	0.44	2.76
GGG G	1.12	0.92	2.07	0.52	AGC S	0.71	0.82	3.03	0.25
GGU G	2.10	1.42	0.67	2.63	AGU S	1.26	1.24	0.27	4.63
CAC H	0.89	1.21	1.89	0.56	UCA S	1.48	1.78	0.43	3.79
CAU H	1.26	1.87	0.52	3.01	UCC S	0.98	1.34	1.33	0.87
**AUA I**	**2.44**	**2.64**	0.20	**12.70**	UCG S	1.10	1.38	1.05	1.18
AUC I	2.00	1.80	1.68	1.13	UCU S	1.88	1.69	0.51	3.5
AUU I	2.87	2.88	0.44	6.53	ACA T	2.01	1.87	0.61	3.18
**AAA K**	**4.70**	**3.76**	0.25	**16.92**	ACC T	1.32	1.38	1.98	0.68
**AAG K**	**2.48**	1.91	2.53	**0.87**	ACG T	1.62	1.57	1.29	1.24
CUA L	0.85	0.93	0.27	3.30	ACU T	1.57	1.31	0.39	3.69
CUC L	1.26	1.09	1.47	0.80	GUA V	1.80	1.54	0.25	6.68
CUG L	0.85	1.08	6.85	0.14	GUC V	1.35	1.25	1.16	1.12
CUU L	1.65	1.67	0.50	2.00	GUG V	1.57	1.37	4.23	0.35
**UUA L**	1.39	**1.81**	0.06	**26.67**	**GUU V**	**2.34**	2.28	0.40	**5.78**
**UUG L**	**1.76**	**2.06**	0.56	**3.41**	UGG W	1.09	1.24	1.61	0.72
AUG M	2.76	1.97	1.84	2.04	**UAC Y**	**1.52**	**1.46**	1.42	**1.05**
					UAU Y	2.24	2.66	0.38	6.45

^1^ Ratio of the average codon usage of the two viruses divided by codon usage of the host *C. variabilis*. Ratios above 1 favor the virus and ratios less than 1 favor the host. Codons in bold font are recognized by tRNAs encoded by one or both chloroviruses.

**Table 6:** Total tRNA genes and clusters of representative *Phycodnaviridae* viruses ^a^.

Phycodnaviridae Viruses	Accession Number	Total tRNA Genes	tRNA Genes in Cluster ^b^
Bathycoccus sp. RCC1105 virus BpV1	HM004432.1	2	2
Ectocarpus siliculosus virus 1	AF204951.2	0	0
Emiliania huxleyi virus 86 isolate EhV86	AJ890364.1	4	2
Heterosigma akashiwo virus 01	NC_038553	3	2
Micromonas pusilla virus 12T	NC_020864.1	6	5
Micromonas sp. RCC1109 virus MpV1	HM004429.1	6	3
Only Syngen Nebraska virus 5	KX857749.1	14	14
Ostreococcus lucimarinus virus OlV1	HM004431.1	4	4
Ostreococcus lucimarinus virus 2 isolate Olv2	KP874736.1	5	4
Ostreococcus lucimarinus virus 7 isolate OlV7	KP874737.1	4	4
Ostreococcus mediterraneus virus 1 isolate OmV1	KP874735.1	3	3
Ostreococcus tauri virus 2	FN600414.1	3	3
Ostreococcus tauri virus OtV5	EU304328.2	3	3
Yellowstone lake phycodnavirus 1	LC015647.1	7	2 and 5 ^c^
Yellowstone lake phycodnavirus 2	LC015648.1	4	4
Yellowstone lake phycodnavirus 3	LC015649.1	4	3

^a^ Data for this table were obtained from Morgado and Vincenta [1]. ^b^ Definition of a cluster: no intervening genes; 2 or more to be considered as a cluster. ^c^ There are 2 tRNA clusters, one with 2 and the other with 5; tRNA genes 2 and 3 are separated by 360 nt that encodes a putative protein.

**Table 7:** Total tRNA genes and clusters of representative large DNA viruses ^a,b^.

Ascoviridae	Accession Number	Total tRNA Genes	tRNA Genes in Cluster ^c^
Heliothis virescens ascovirus 3f isolate LD135790	KJ755191.1	1	0
Heliothis virescens ascovirus 3g	JX491653.1	1	0
Trichoplusia ni ascovirus 2c	DQ517337.1	3	0
**Asfarviridae**			
African swine fever virus strain Ken06.Bus	NC_044946	0	0
African swine fever virus strain BA71V	NC_001659	0	0
African swine fever virus isolate ASFV Belgium 2018/1	LR536725.1	0	0
**Faustoviruses**			
Faustovirus strain E9	MT335755	0	0
Faustovirus strain D3	KU556803	0	0
Faustovirus strain E24	KU702948.1	0	0
**Iridoviridae**			
Aedes taeniorhynchus iridescent virus,	DQ643392.1	1	0
Lymphocystis disease virus—isolate China	AY380826.1	1	0
Scale drop disease virus isolate C4575	KR139659.1	1	0
Singapore grouper iridovirus	AY521625.1	1	0
**Kaumeobavirus**			
Kaumeobavirus isolate Sc	NC_034249.1	0	0
Kaumoebavirus isolate Sc	KX552040.1	0	0
**Marseilliviridae**			
Brazilian marseillevirus strain BH2014	NC_029692.1	0	0
Golden Marseillevirus	NC_031465.1	0	0
Marseillevirus strain T19	NC_013756	0	0
**Mimiviridae**			
Acanthamoeba polyphaga mimivirus	HQ336222.2	6	2
Acanthamoeba polyphaga moumouvirus	JX962719.1	2	2
Aureococcus anophagefferens virus isolate BtV-01	KJ645900.1	7	5
Cafeteria roenbergensis virus BV-PW1	GU244497.1	22	22
Chrysochromulina ericina virus isolate CeV-01B	KT820662.1	12	3 and 7 ^d^
Megavirus chiliensis	JN258408.1	3	0
Mimivirus terra2	KF527228.1	6	2
Phaeocystis globosa virus strain 16T	KC662249.1	8	7
Tetraselmis virus 1	KY322437.1	10	10
**Molliviruses**			
Mollivirus kamchatka strain Kronotsky	MN812837.1	0	0
Mollivirus sibericum isolate P1084-T	NC_027867.1	2	0
Mollivirus sibericum isolate P1084-T	KR921745.1	3	0
**Orpheovirus**			
Orpheovirus IHUMI-LCC2	NC_036594.1	0	0
**Pacmanvirus**			
Pacmanvirus A23	NC_034383.1	0	0
**Pandoraviruses**			
Pandoravirus macleodensis	NC_037665	1	0
Pandoravirus neocaledonia	NC_037666	3	0
Pandoravirus quercus	NC_037667	1	0
**Pithoviridae**			
Brazilian cedratvirus IHUMI strain IHUMI-27.7	LT994651	0	0
Cedratvirus kamchatka isolate P4	MN873693.1	0	0
Pithovirus sp. LC8	LT598836	0	0
**Poxviridae**			
Parapoxvirus red deer/HL953 strain HL953	KM502564.1	1	0
Squirrelpox virus Berlin_2015 (unverified)	MF503315.1	1	0
**Baculoviridae**			
Anticarsia gemmatalis multicapsid nucleopolyhedrovirus isolate AgMNPV-37	KR815466.1	1	0
Anticarsia gemmatalis nucleopolyhedrovirus	DQ813662.2	1	0
Autographa californica nucleopolyhedrovirus clone C6	L22858.1	1	0
Neodiprion lecontei NPV	AY349019.1	1	0
Antheraea pernyi nucleopolyhedrovirus	DQ486030.3	1	0
Helicoverpa armigera granulovirus	EU255577.1	1	0
Mamestra brassicae MNPV strain K1	JQ798165.1	1	0
Mamestra configurata NPV-A strain 90/2	U59461.2	1	0
Xestia c-nigrum granulovirus	AF162221.1	1	0
**Herpesviridae**			
Bovine herpesvirus type 1.1	AJ004801.1	2	0
Cercopithecine herpesvirus 16 strain X313	DQ149153.1	2	0
Columbid alphaherpesvirus 1 strain HLJ	KX589235.1	19	0
Falconid herpesvirus 1 strain S-18	KJ668231.1	19	0
Macropodid herpesvirus 1 isolate MaHV1.3076/08	KT594769.1	1	0
Murine herpesvirus 68 strain WUMS	U97553.2	7	4 and 2 ^e^

^a^ Data for this table were obtained from Morgado and Vincente [1] and NCBI. ^b^ Table does not include the *Phycodnaviridae*. ^c^ Definition of a cluster: no intervening genes; 2 or more to be considered as a cluster. ^d^ There are 2 tRNA clusters, one with 3 and the other with 7. ^e^ There are 2 tRNA clusters, one with 4 and the other with 2.

## Supplementary Materials

The following are available online at https://www.mdpi.com/1999-4915/12/10/1173/s1, Figure S1: Alignment of tRNA^Thr^ genes from 13 SAG viruses, Figure S2: Phylogenetic tree of tRNA^Tyr^ genes from 35 chloroviruses representing all three clades, NC64A, Pbi and SAG, Figure S3: Phylogenetic tree of tRNA^Gly^ genes from 39 chloroviruses representing all three clades, NC64A, Pbi and SAG, Figure S4: Phylogenetic tree of tRNA^Arg^ genes from all 41 chloroviruses representing all three clades, NC64A, Pbi and SAG. Table S1: Accession numbers for 41 chloroviruses grouped into three clades that have three different hosts, Table S2: NC64A viruses: 5’ and 3’ genes closest to the tRNA gene cluster, Table S3: SAG viruses: 5’ and 3’ genes closest to the tRNA gene cluster, Table S4: Pbi viruses: 5’ and 3’ genes closest to the tRNA gene cluster.
