# Supplementary material for: Diversity of tRNA Clusters in the Chloroviruses
Source: Viruses. 2020 Oct 16;12(10):1173. doi: 10.3390/v12101173 (PMC7589089; doi:10.3390/v12101173)
Supplement: Supplementary file 1 [file viruses-12-01173-s001.pdf]

viruses

MDPI

Article

Diversity of tRNA Clusters in the Chloroviruses

Garry A. Duncan [1], David D. Dunigan [1,2] and James L. Van Etten[1,2,*]

[1] Nebraska Center for Virology, University of Nebraska-Lincoln, Lincoln, NE 68583-0900, USA; gduncan2@unl.edu (G.A.D.); ddunigan2@unl.edu (D.D.D.); jvanetten1@unl.edu (J.L.V.E.)
[2] Department of Plant Pathology, University of Nebraska-Lincoln, Lincoln, NE 68583-0833, USA
* Correspondence: jvanetten1@unl.edu; Tel.: (+1)-402-472-3168

Supplementary Material

Table S1. Accession numbers for 41 chloroviruses grouped into three clades that have three different hosts.

Chloro-viruses	Accession number	Sampling Location[1]	Chlorovirus group
MA-1E	JX997173	Massachussetts, USA	NC64A
CvsA1	JX997165	Sawara, Japan	NC64A
CviKI	JX997162	Kyoto, Japan	NC64A
KS1B	JX997171	Kansas, USA	NC64A
PBCV-1	JF411744.1	North Carolina, USA	NC64A
IL-3A	JX997169	Illinois, USA	NC64A
MA-1D	JX997172	Massachussetts, USA	NC64A
NE-JV-4	JX997179	Nebraska, USA	NC64A
AN69C	JX997153	Canberra, Australia	NC64A
NY-2B	JX997182	New York state, USA	NC64A
IL-5-2s1	JX997170	Illinois, USA	NC64A
NY-2A	DQ491002.1	New York state, USA	NC64A
NYs-1	JX997183	New York state, USA	NC64A
AR158	DQ491003.2	Buenos Aires, Argentina	NC64A
Fr5L	JX997167	France	Pbi
CZ-2	JX997166	Czech Republica	Pbi
MT325	DQ491001.1	Montana, USA	Pbi
Can18-4	JX997157	Canada	Pbi
CVB-1	JX997160	Berlin, Germany	Pbi
FR483	DQ890022.1	France	Pbi
CVG-1	JX997161	Göttingen, Germany	Pbi
CVR-1	JX997164	Rauschenberg, Germany	Pbi
CVA-1	JX997159	Amönau, Germany	Pbi
AP110	JX997154	Unknown	Pbi
CVM-1	JX997163	Marburg, Germany	Pbi
NW665.2	JX997181	Norway	Pbi
OR0704.2.2	JX997184	Oregon, USA	Pbi
NE-JV-1	JX997176	Nebraska, USA	Pbi
OR0704.3	JX997185	Oregon, USA	SAG
Can0610SP	JX997156	British Columbia, Canada	SAG
NE-JV-2	JX997177	Nebraska, USA	SAG
NE-JV-3	JX997178	Nebraska, USA	SAG
ATCV-1	EF101928	Stuttgart, Germany	SAG
WI0606	JX997187	Wisconsin, USA	SAG
MO0605SPH	JX997175	Missouri, USA	SAG
GM0701.1	JX997168	Guatemala	SAG
Br0604L	JX997155	SaoPaolo, Brazil	SAG
TN603.4.2	JX997186	Tennessee, USA	SAG
Canal-1	JX997158	Nebraska, USA	SAG

MN0810.1	JX997174	Minnesota, USA	SAG
NTS-1	JX997180	Nebraska, USA	SAG

[1]Sampling locations obtained from Jeanniard et al. [13].

viruses

Table S2. NC64A viruses: 5' and 3' genes closest to the tRNA gene cluster.

NC64A virus	5' upstream (~1068 nt)	5' upstream (~291 nt)	tRNA cluster	3' downstream (~1299 nt)	3' downstream (~1140)	3' downstream[1] (~1191 nt)
**MA-1E**	390L	**393R**			**396R**	407L
**CvsA1**	361L	**364R**			**368R**	380L
**CviKI**	353L	**356R**			**359R**	370L
**KS1B**	310L	**311R**				**314L**
**PBCV-1**	A328L	**A329R**		**A330R**		A333L
**IL-3A**	368L	**369R**		**371R**	375R	386L
**MA-1D**	**347L**				**355R**	367L
**NE-JV-4**	384L	**385R**		**388R**		390L
**AN69C**	377L	**378R**		**380R**	384R	395L
**NY-2B**	**465L**				473R	484L
**IL-5-2s1**	**484L**				**492R**	503/506L
**NY-2A**	B458L	**B460R**			B465R	B480L
**NYs-1**	**474L**				**483R**	495L
**AR158**	**C406L**				C413R	C423L

The genes in each column are orthologs to one another; approximate nt length of each ortholog is noted in parentheses. The genes in bold font are the closest 5' and 3' genes for each of the 14 NC64A viruses. There is a common tRNA cluster location among the NC64A viruses. Red column heading and column are used to indicate the position of the tRNA clusters in the viral genomes relative to the surrounding non-tRNA genes.

[1]This column is included because the KS1B gene 314L is the closest 3' gene. Orthologs of 314L are present in the other NC64A, but they are further downstream.

Table S3. SAG viruses: 5′ and 3′ genes closest to the tRNA gene cluster.

SAG viruses	5' upstream (~285 nt)	5' upstream (~837 nt)	tRNA cluster	3' downstream (~1041 nt)	3' downstream (~3,774 nt)
**OR0704.3**	**307R**				**301L**
**Can0610SP**	**308R**			**309R**	313L
**NE-JV-2**	338R	**339R**			**341L**
**NE-JV-3**	**301R**				**303L**
**ATCV-1**	**Z254R**				**Z257L**
**WI0606**	**329R**				**332L**
**MO0605SPH**	**313R**				**316L**
**GM0701.1**	**305R**			**309R**	312L
**Br0604L**	**306R**				**308L**
**TN603.4.2**	**303R**				**307L**
**Canal-1**	**302R**				**304L**
**MN0810.1**	**337R**				**340L**
**NTS-1**	**345R**				**351L**

The genes in each column are orthologs to one another; approximate nt length of each ortholog is noted in parentheses. The genes in bold font are the closest 5' and 3' genes for each of the 13 SAG viruses. There is a common tRNA cluster location among the SAG viruses. Red column heading and column are used to indicate the position of the tRNA clusters in the viral genomes relative to the surrounding non-tRNA genes.

viruses

MDPI

Table S4. Pbi viruses: 5' and 3' genes closest to the tRNA gene cluster.

**Pbi viruses**	**5' upstream (~726 nt)**	**5' upstream (~270 nt)**	**5' upstream (870 nt)**	**5' upstream (267 nt)**	**tRNA cluster**	**3' downstream (789 nt)**	**3' downstream (~297 nt)[1]**	**3' downstream (~420 nt)**	**3' downstream (~552 nt)**
**Fr5L**	393R	**397R**						**401L**	
**CZ-2**	352R	**355R**						**358L**	
**MT325**	**M3422R**						**M344L**		
**Can18-4**	**414R**				418R		**419L**	422L	
**CVB-1**	**406R**				408R		**411L**	413L	
**FR483**	**N351R**						**N345L**	n356L	
**CVG-1**	**385R**						**390L**	392L	
**CVR-1**	**400R**						**404L**	406L	
**CVA-1**	**392R**						**396L**	398L	
**AP110A**	**403R**					**407R**	411L	413L	
**CVM-1**	**421R**						**425L**	428L	
**NW665.2**	**375R**				378R		**381L**	383L	
**OR0704.2.2**	349R	352R	**353R**					**356L**	
**NE-JV-1**				**683R**	688R				**690L**

The genes in each column are orthologs to one another; approximate nt length of each ortholog is noted in parentheses. There is a common tRNA cluster location among the Pbi viruses, with the exception of NE-JV-1. Several viruses have protein-encoding genes within the tRNA cluster (blue font), which are not orthologous to one another. The HGT events that led to these gene insertions resulted in the loss of one or more tRNA genes in each case. Red column heading and column are used to indicate the position of the tRNA clusters in the viral genomes relative to the surrounding non-tRNA genes.

[1]MT325 and FR483 have longer genes (525 nt) but the core of 297 is present with high identity

viruses

MDPI

```
                      Box A                                             Box B
                   TRGYNNARNNG                                       RGTTCRANTCC
ATCV1:     GCTCCCATAGCTCAGTTGGTTAGAGCGCGACTTTAGTAAGGTCGAGGTCCGTGGTTCGAATCCGCGTGGGAGCA
WI0606:    GCTCCCATAGCTCAGTTGGTTAGAGCGCGACTTTAGTAAGGTCGAGGTCCGTGGTTCGAATCCGCGTGGGAGCA
OR0704.3:  GCTCCCATAGCTCAGTTGGTTAGAGCGCGACTTTAGTAAGGTCGAGGTCCGTGGTTCGAATCCGCGTGGGAGCA
NTS-1:     GCTCCCATAGCTCAGTTGGTTAGAGCGCGACTTTAGTAAGGTCGAGGTCCGTGGTTCGAATCCGCGTGGGAGCA
NE-JV-3:   GCTCCCATAGCTCAGTTGGTTAGAGCGCGACTTTAGTAAGGTCGAGGTCCGTGGTTCGAATCCGCGTGGGAGCA
NE-JV-2:   GCTCCCATAGCTCAGTTGGTTAGAGCGCGACTTTAGTAAGGTCGAGGTCCGTGGTTCGAATCCGCGTGGGAGCA
MO0605SH:  GCTCCCATAGCTCAGTTGGTTAGAGCGCGACTTTAGTAAGGTCGAGGTCCGTGGTTCGAATCCGCGTGGGAGCA
Can0610SP: GCTCCCATAGCTCAGTTGGTTAGAGCGCGACTTTAGTAAGGTCGAGGTCCGTGGTTCGAATCCGCGTGGGAGCA
TN703.4.2: GCTCCCATAGCTCAGATGGTTAGAGCGCGACTTTAGTAAAGTCGAGGTCCGTGGTTCGAATCCGCGTGGGAGCA
GM0701.1:  GCTCCCATAGCTCAGATGGTTAGAGCGCGACTTTAGTAAAGTCGAGGTCCGTGGTTCGAATCCGCGTGGGAGCA
Br0604L:   GCTCCCATAGCTCAGATGGTTGGAGCGCGACTTTAGTAAGATCGAGGTCCGTGGTTTGAATCCGCGTGGGAGCA
MN0810.1:  GCTCCTATAGCTCAGTGGA--AGAGCGCGACTTTAGTAAGGTCGAGGCCCGTGGTTCGACCCCACGTGGGAGCA
Canal-1:   GCTCCTATAGCTCAGCGG--TAGAGCGCGACTTTAGTAAGGTCGAGGTCCATGGTTC-AACCCGTGTGGGAGCA
```

Figure S1. Alignment of $tRNA^{Thr}$ genes from 13 SAG viruses. The two rectangles, known as Box A and B, are recognized by transcription factor IIIC, critical to the eventual attachment of RNA polymerase III. The eukaryotic consensus sequences reported by Diebel et al. [28] are above the rectangles. The 5′ to 3′ anticodon in identified in bold font. Invariant bases for Box A and B are highly conserved. The $tRNA^{Thr}$ genes for the SAG viruses are orphaned from the tRNA clusters, but their high level of sequence identity suggests that they are transcribed by RNA polymerase III independent of their respective tRNA clusters.

viruses MDPI

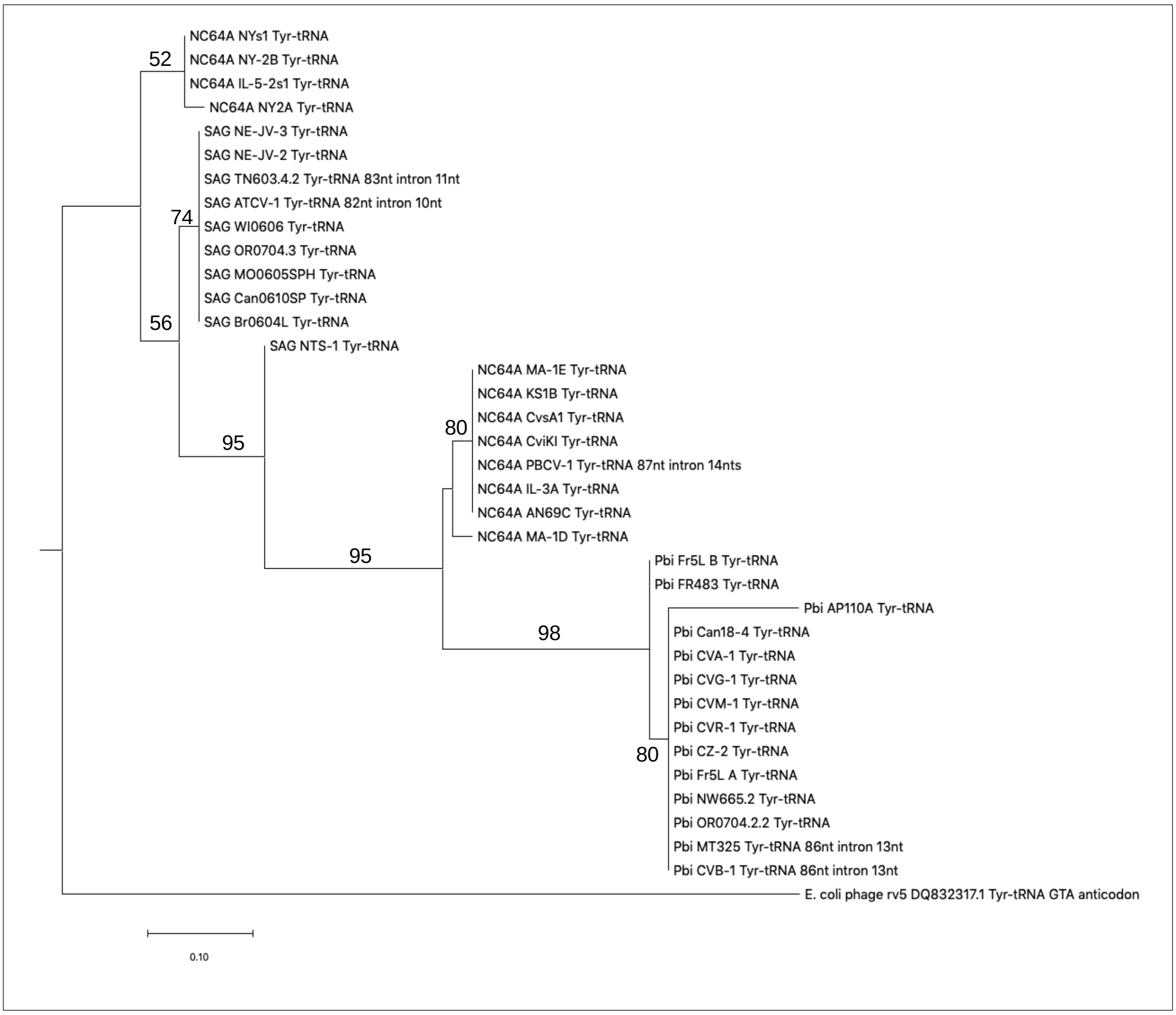

Figure S2. Phylogenetic tree of tRNA^{Tyr} genes from 35 chloroviruses representing all three clades, NC64A, Pbi and SAG. Six chloroviruses lacked the tRNA^{Tyr} gene. The tRNA^{Tyr} gene from *E. coli* phage rv5 was used as the outgroup. One subclade of NC64A viruses is more similar to SAG viruses, while the other subclade of NC64A viruses is more similar to the Pbi viruses. Bootstrap values greater than 50 are reported. The sequences were aligned with MUSCLE and the trees were constructed using the maximum.

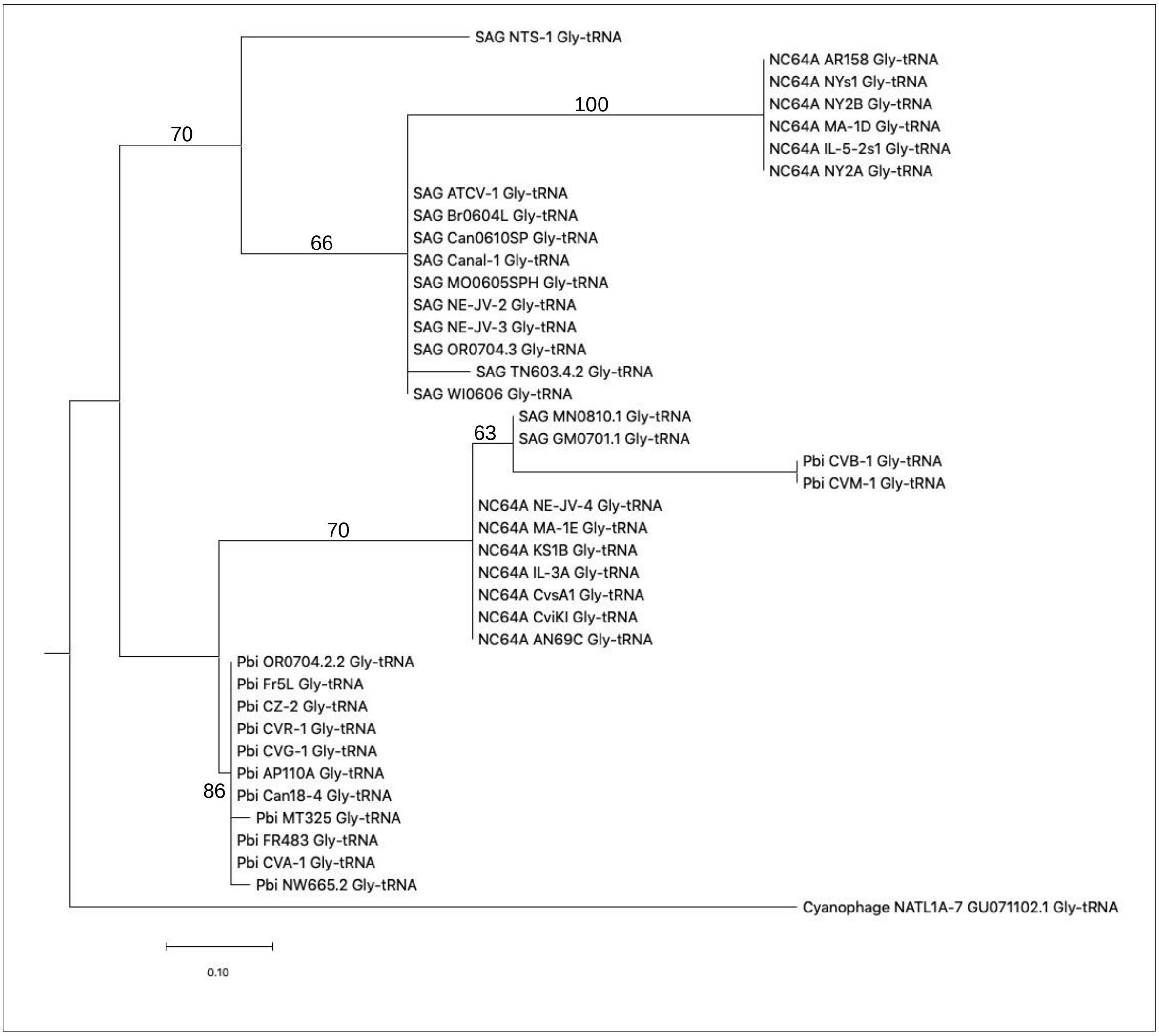

Figure S3. Phylogenetic tree of $tRNA^{Gly}$ genes from 39 chloroviruses representing all three clades, NC64A, Pbi and SAG. Two chloroviruses lacked the $tRNA^{Gly}$ gene. The $tRNA^{Gly}$ gene from cyanophage NATL1A was used as the outgroup. One NC64A subclade is more similar to SAG viruses than the other subclade of NC64A viruses. Two SAG viruses and two Pbi viruses are more similar to a second subclade of NC64A than they are to viruses in their own clades. Bootstrap values greater than 50 are reported. The sequences were aligned with MUSCLE and the trees were constructed using the maximum likelihood algorithm.

viruses

MDPI

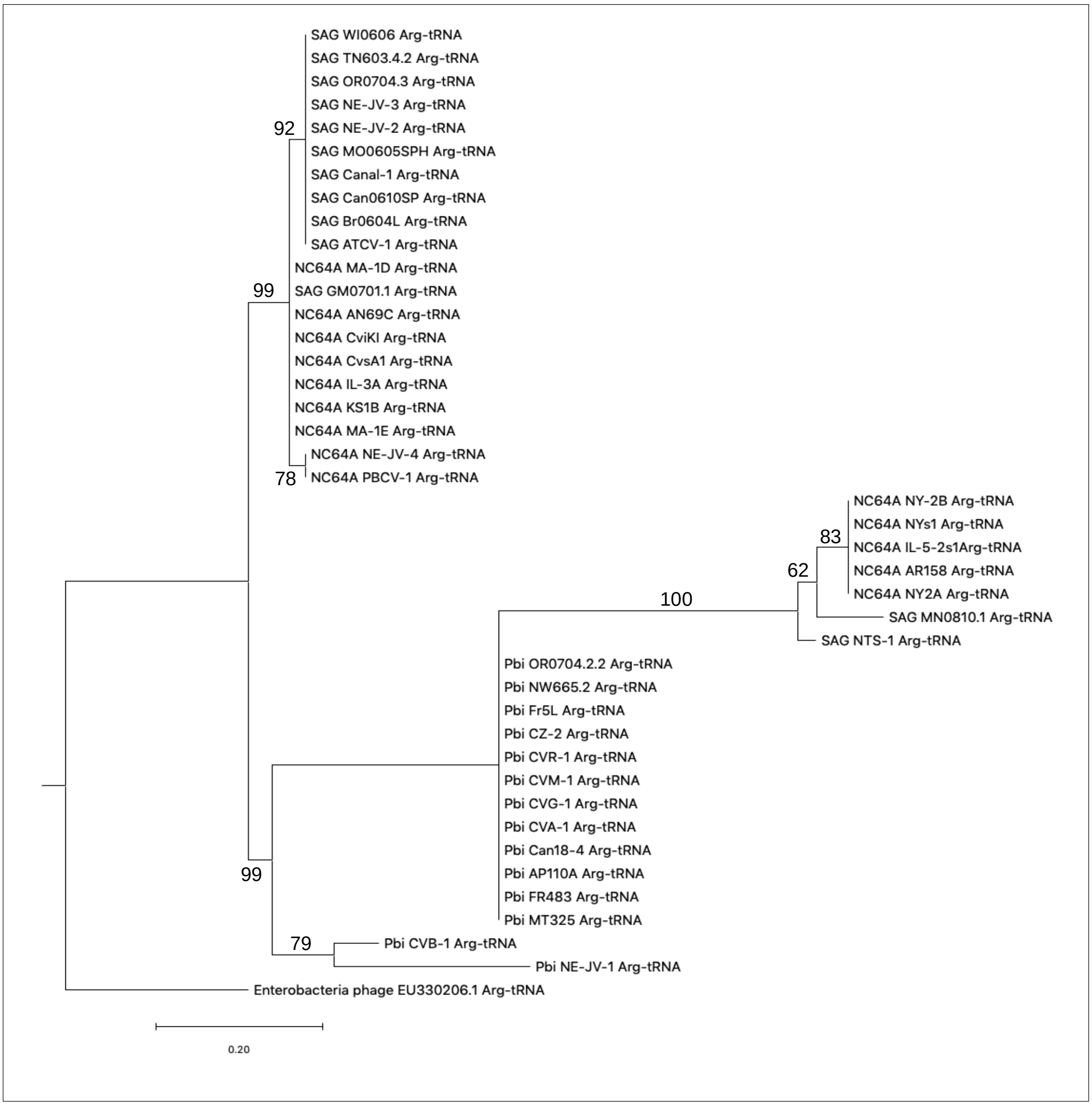

Figure S4. Phylogenetic tree of $tRNA^{Arg}$ genes from all 41 chloroviruses representing all three clades, NC64A, Pbi and SAG. The $tRNA^{Arg}$ gene from enterobacteria phage EU330206.1 was used as the outgroup. One NC64A subclade and two SAG viruses are more similar to Pbi viruses than they are to viruses in their own clade. Bootstrap values greater than 50 are reported. The sequences were aligned with MUSCLE and the trees were constructed using the maximum likelihood algorithm.

© 2020 by the authors. Submitted for possible open access publication under the terms and conditions of the Creative Commons Attribution (CC BY) license (http://creativecommons.org/licenses/by/4.0/).
